# Pan-Genome Analysis Links the Hereditary Variation of *Leptospirillum ferriphilum* With Its Evolutionary Adaptation

**DOI:** 10.3389/fmicb.2018.00577

**Published:** 2018-03-27

**Authors:** Xian Zhang, Xueduan Liu, Fei Yang, Lv Chen

**Affiliations:** ^1^Department of Occupational and Environmental Health, Xiangya School of Public Health, Central South University, Changsha, China; ^2^School of Minerals Processing and Bioengineering, Central South University, Changsha, China; ^3^Key Laboratory of Biometallurgy of Ministry of Education, Central South University, Changsha, China

**Keywords:** *Leptospirillum ferriphilum*, mathematical models, pan-genome, hereditary variation, adaptive evolution

## Abstract

Niche adaptation has long been recognized to drive intra-species differentiation and speciation, yet knowledge about its relatedness with hereditary variation of microbial genomes is relatively limited. Using *Leptospirillum ferriphilum* species as a case study, we present a detailed analysis of genomic features of five recognized strains. Genome-to-genome distance calculation preliminarily determined the roles of spatial distance and environmental heterogeneity that potentially contribute to intra-species variation within *L. ferriphilum* species at the genome level. Mathematical models were further constructed to extrapolate the expansion of *L. ferriphilum* genomes (an ‘open’ pan-genome), indicating the emergence of novel genes with new sequenced genomes. The identification of diverse mobile genetic elements (MGEs) (such as transposases, integrases, and phage-associated genes) revealed the prevalence of horizontal gene transfer events, which is an important evolutionary mechanism that provides avenues for the recruitment of novel functionalities and further for the genetic divergence of microbial genomes. Comprehensive analysis also demonstrated that the genome reduction by gene loss in a broad sense might contribute to the observed diversification. We thus inferred a plausible explanation to address this observation: the community-dependent adaptation that potentially economizes the limiting resources of the entire community. Now that the introduction of new genes is accompanied by a parallel abandonment of some other ones, our results provide snapshots on the biological fitness cost of environmental adaptation within the *L. ferriphilum* genomes. In short, our genome-wide analyses bridge the relation between genetic variation of *L. ferriphilum* with its evolutionary adaptation.

## Introduction

The emergence of next generation sequencing technologies accompanied with developing methodological and computational approaches has yielded valuable insights into the genetic traits of microorganisms in various habitats worldwide, such as their metabolic capabilities and evolutionary adaptation (Cordero and Polz, 2014; Ji et al., 2014; Zhang and Sievert, 2014; Youssef et al., 2015; Zhang et al., 2016a,b,e, 2017a). Plentiful genome data deluge in public databases have fueled the field of comparative genomics. Studies on bacterial genomes significantly expanded the scope of inter-species divergence (Ullrich et al., 2016; Zhang et al., 2016e). Also, intra-species differentiation has been observed based on individual genomes of certain species (Zhang et al., 2016a,b, 2017a). Spatial distance and environmental heterogeneity are recognized to be two major factors that contribute to genetic variation of microbial genomes and populations (Ramette and Tiedje, 2007). At the spatial scale, the contribution of environmental factors to microbial biogeography is relatively more than that of geographic distributions (Lin et al., 2013). As such, it is of value to determine the potential relevance between hereditary variation of bacterial strains and adaptation to different ecological niches, probably reflecting the responsive mechanisms to local environmental perturbations.

Novel genes, in theory, would be added to the genome of the species after new genomes are sequenced (Medini et al., 2005), thereby expanding microbial gene pool. The coinage ‘pan-genome’ (‘pan,’ derived from the Greek word ‘*παν*,’ meaning ‘whole’) was first introduced a decade ago (Tettelin et al., 2005) in order to delineate the intra-species diversity. Pan-genome analysis provides a framework not only to estimate the genomic diversity by means of the dataset at hand, but also to predict, via mathematical extrapolation based on sufficient samples (at least five genomes; Vernikos et al., 2015), the number of additional whole genomes that are necessary to fully characterize the entire gene repertoire of a given species. Bacterial pan-genome is composed of ‘core genome’ containing genes shared by all strains and ‘dispensable genome’ containing genes shared by a subset of the strains and the strain-specific genes (Medini et al., 2005; Tettelin et al., 2008). Core genome encodes biological functions that are essential to basic lifestyle and phenotypes, while dispensable genome was responsible for species diversity and probably contributes to the selective advantages, such as econiche adaptation. The flexible gene pool endows microorganisms with strain-specific adaption to local environmental conditions (Acuña et al., 2013). Accordingly, it is of interest to estimate the sizes of pan-genome, core genome, and new genes of a given species as novel genomes are added, and further identify the relative contribution of dispensable genome to inheritance variation and its relatedness with specific adaptation to environmental niches.

*Leptospirillum* spp. are Gram-negative, vibrio- or spiral-shaped, and obligately chemolithotrophic bacteria (Coram and Rawlings, 2002), which are phylogenetically affiliated with the deep branching class *Nitrospira* (Bonnefoy and Holmes, 2012; Goltsman et al., 2013). They ubiquitously occur in a variety of acidophilic microbial communities (Zhang et al., 2016c), and are recognized to be the critical biological catalysts in both natural and deliberate metal sulfide biooxidation processes (Coram and Rawlings, 2002; Chen et al., 2013). Species of *Leptospirillum* genus are the dominant iron-oxidizing bacteria in metal-tolerant, acidophilic microbial consortia that prompt ferric iron [Fe(III)]-mediated oxidative dissolution of sulfide minerals, suggesting their key roles in the biogeochemical cycle of iron. Under the environmental conditions characterized by temperature above 40°C and pH value below 1.0, leptospirilla have been reported to be the principal contributors responsible for the formation of acid mine drainage (Sand et al., 1992; Schrenk et al., 1998; Coram and Rawlings, 2002).

Considerable variation among *Leptospirillum* isolates has been exhibited in previous studies (Harrison and Norris, 1985). To date, four known groups within *Leptospirillum* clade are group I (*L. ferrooxidans*), group II (*L. ferriphilum* and *L. rubarum*), group III (*L. ferrodiazotrophum*), and group IV (Hippe, 2000; Coram and Rawlings, 2002; Tyson et al., 2005; Goltsman et al., 2013; Zhang et al., 2016c). Each is an obligately chemolithotrophic organism capable of assimilation of inorganic form of carbon, solely deriving energy from aerobic oxidation of iron (Hippe, 2000; Coram and Rawlings, 2002). In *Leptospirillum* groups, a diazotrophic lifestyle has been previously documented (Parro and Moreno-Paz, 2004; Tyson et al., 2005; Goltsman et al., 2009, 2013; Galleguillos et al., 2011, 2013). Of all leptospirilla, *L. ferriphilum* (*ferri*, iron; *philum*, loving) has been proposed to be a separate species, which was clearly distinguished from *L. ferrooxidans* isolates by means of a 16S rRNA phylogeny (Coram and Rawlings, 2002). In their study, some efforts were invested in order to delineate certain key phenotypes of *L. ferriphilum* in the aspects of its physiological and physical properties, such as nutritional type, cell shape, and optimum conditions for bacterial growth.

Recently, several *L. ferriphilum* genomes are available in public database, owing to the implementation of high-throughput sequencing technologies. Much research has focused on individual genomes of *L. ferriphilum* isolates in various ecological environments, yet relatively little is known about their phylogenetic differentiation. In this study, we therefore selected a total of five distinct strains (DX, ZJ, ML-04, YSK, and Sp-Cl) for comparative survey. We present the comprehensive study of *L. ferriphilum* pan-genome and the elucidation of genetic diversity among *L. ferriphilum* strains. Our results shed light on the prevalence of horizontal gene transfer (HGT) events, accompanied by genome reduction, and are conducive to elaborating the potential relevance between hereditary differentiation driven by gene gain and/or loss and evolutionary adaption of *L. ferriphilum* genomes.

## Materials and Methods

### Bacterial Genomes Used in This Study

Five *L. ferriphilum* genomes available in NCBI repository were collected for this study, including the draft genomes of strains DX and ZJ isolated from two different copper mine tailings in China, the complete genome (NCBI ID: CP002919) of strain ML-04 obtained from an acidic water near a hot spring in China (Mi et al., 2011), the complete genome (NCBI ID: CP007243) of strain YSK isolated from an acid mine drainage in China (Jiang et al., 2015), and the draft genomes of strain Sp-Cl obtained from a bioleaching solutions draining in Chile (Issotta et al., 2016). However, genome of strain DSM 14647 (Cárdenas et al., 2014) was excluded in our study due to the relatively low values of BLASTN-based average nucleotide identity (ANI; <95%) and tetranucleotide composition regression (TETRA; <0.99) with other available *L. ferriphilum* genomes in the public database, which were calculated by the software JSpecies v1.2.1 (Richter and Rosselló-Móra, 2009) (unpublished data). General features of bacterial genomes used in this study were summarized in **Table 1**. Herein, the quality of microbial genomes was evaluated by the CheckM package (Parks et al., 2015) with the default parameters.

**Table 1:** Genome characteristics of *Leptospirillum ferriphilum* isolates from various acidic environments worldwide.

Feature	Strains within the *Leptospirillum ferriphilum* species				
	DX	ZJ	ML-04	YSK	Sp-Cl
Geographic origin	Bioleaching heap, Jiangxi, China	Bioleaching heap, Fujian, China	Acidic water near a hot spring, Tengchong, China	Acid mine drainage, Jiangxi, China	Bioleaching solutions draining, Atacama Desert, Chile
Accession number	MPOJ00000000	MPOK00000000	CP002919	CP007243	LGSH00000000
Genome size (Mb)	2.36	2.34	2.41	2.33	2.48
Genome status	Draft	Draft	Complete	Complete	Draft
Coverage (×)	165	52			
Completeness (%)^∗^	93.15	93.02	—	—	90.42
GC content (%)	54.50	54.70	54.60	54.50	54.40
# Contigs	30	104	1	1	74
Protein-coding sequences	2,324	2,312	2,378	2,273	2,419
rRNA operons	1	1	2	2	1
tRNA genes	49	48	53	52	48
IS elements	69	63	106	74	64
COG	1,695 (72.9%)	1,713 (74.1%)	1,746 (73.4%)	1,689 (74.3%)	1,714 (70.9%)
Reference	Zhang et al., 2017b	Zhang et al., 2017b	Mi et al., 2011	Jiang et al., 2015	Issotta et al., 2016

*^∗^The genome completeness was calculated using CheckM (Parks et al., 2015). Strains ML-04 and YSK were not included since their genomes were complete.*

### Genome-to-Genome Distance Calculation

Genome sequence-based classification of microorganisms underlying genome Blast distance phylogeny has been recognized to be a digital DNA–DNA hybridization (DDH) replacement (Meier-Kolthoff et al., 2013). In this study, an updated and enhanced platform Genome-to-Genome Distance Calculator (GGDC) v2.1^1^ with improved DDH-prediction models and a set of novel features such as confidence-interval estimation was employed to calculate the intergenomic distances between pairs of entirely sequenced genomes. Distance values *d*(*X, Y*) between genomes X and Y were calculated according to the following formulae:

(1) $$\begin{array}{c} d_{1}(X,Y)\;=\;1-\frac{H_{XY}+H_{XY}}{\lambda (X,Y)}\;\left(1\right) \end{array}$$

(2) $$\begin{array}{c} d_{2}(X,Y)\;=\;1-\frac{2\cdot I_{XY}}{\lambda (X,Y)}\;\left(2\right) \end{array}$$

in which, *XY* denotes BLAST run using subject genome X and query genome Y, *H_XY_* represents the total length of all high-scoring segment pairs (HSPs) between both genomes, λ(*X,Y*) indicates the sum of both genomes’ lengths, and *I_XY_* means the sum of identical base pairs over all HSPs. An easy-to-use toolkit HemI for heatmaps (Deng et al., 2014) was then used to visualize the distance values.

### Pan-Genome Analysis of *L. ferriphilum* Species

In this protocol, entire protein sequences were first extracted using in-house Perl scripts. In order to determine the orthologous clusters among these five strains, a BLASTP all-versus-all pairwise comparison of the complete proteomes was performed to identify Best Bidirectional Blast Hit (BBBH). The determination of BBBH was based on the BLAST program with *E*-value threshold of 1e^-5^ and sequence identity cut-off of 50%. Of note, predicted MGEs were excluded, given that they might interfere with the results due to lineage-specific expansions (Carretero-Paulet et al., 2015). The orthologous clusters were classified into core-, dispensable-, and unique-genomes implementing the program PanOCT v3.18 (Fouts et al., 2012) with the following criteria: *E*-value cut-off set to 1e^-5^, sequence identity threshold of 65%, and match length cut-off of 65 bp. The results of pan-genome analysis were manually curated to minimize the possibility of false-negative gene calls. Functional annotation of core genes, dispensable genes, and strain-specific genes was performed using the BLASTP algorithm against the extended Clusters of Orthologous Groups (COG) database (Franceschini et al., 2013) with an *E*-value threshold of 1e^-5^. The COG classification was screened based on the highest hit coverage value as previously described (Ullrich et al., 2016).

### Extrapolation Models for *L. ferriphilum* Pan-Genome

The number of core genes within a given phylogenetic clade and the number of new genes depend on how many bacterial strains are taken into account. As stated by Vernikos et al. (2015), mathematical extrapolation would be robust if sufficient genomes (at least five) are considered. In our study, the sequential inclusion of five *L. ferriphilum* strains within all possible combinations was simulated, as previously described by Tettelin et al. (2005). The size of *L. ferriphilum* pan-genome was extrapolated by fitting the power law regression function *P_s_* = *κn*^γ^, where *P_s_* is the total number of non-orthologous genes within its pan-genome, *n* is the number of sequenced strains, and *κ* and γ are free parameters (Tettelin et al., 2008). The exponent γ < 0 indicates a ‘closed’ pan-genome species since the size of its pan-genome approaches a constant with the increase of bacterial genomes. Conversely, for 0 < γ < 1, species is considered to harbor an ‘open’ pan-genome. In light of the dataset’s normality (Supplementary Figure S1 and Supplementary Table S1), averages of the shared genes were extrapolated implementing an exponential decay function *F_c_* = *κ_c_*exp(-*n*/*τ_c_*) + Ω, where *F_c_* denotes the number of core genes, and *κ_c_, τ_c_*, and Ω are free parameters (Tettelin et al., 2005). In addition, the exponential regression function *F_s_* = *κ_s_*exp(-*n*/*τ_s_*) + *tg*(*𝜃*) was used to model the median sizes of new genes per added genome, where *F_s_* is the number of new genes when the *n*th genome is added, and *κ_s_, τ_s_*, and *tg*(*𝜃*) are free parameters (Tettelin et al., 2005). In the *n*th genome, *N* = 5!/[(*n* – 1)!⋅(5 – *n*)!] represents the number of independent combinations.

### Prediction of Mobile Genetic Elements

Insertion sequences (IS) and transposases distributed over *L. ferriphilum* genomes were predicted and classified using the ISFinder platform (Siguier et al., 2006). A developed IslandViewer 3 (Dhillon et al., 2015), which integrates two sequence composition genomic islands (GIs) prediction method, i.e., IslandPath-DIMOB (Hsiao et al., 2003) and SIGI-HMM (Waack et al., 2006), and a comparative genomic GIs prediction method IslandPick (Langille et al., 2008), was applied for the computational identification of putative GIs. In addition, CRISPR (Clustered Regularly Interspaced Short Palindromic Repeats) loci were identified using the web tool CRISPRFinder (Grissa et al., 2007) or CRISPR Recognition Tool (Bland et al., 2007).

### Comparative Analysis of Architecture and Gene Repertoire of *L. ferriphilum* Genomes

A developed tool Circos (Krzywinski et al., 2009) was used to visualize the similarities and differences of genomic elements arising from BLASTN-based whole genome comparisons. Genomic regions of interest were further analyzed by pairwise comparisons and functional annotation. The entire annotations of targeted genes were subsequently manually checked by sequence alignment against the online server Sequence Similarity Search – BLAST^2^. Pairwise comparisons of specific genomic clusters within *L. ferriphilum* strains were visualized using EasyFig v2.1 (Sullivan et al., 2011). In addition, rRNA and tRNA genes were predicted using the online servers RNAmmer v1.2 (Lagesen et al., 2007) and tRNAscan-SE v2.0 (Lowe and Eddy, 1997), respectively.

### Availability of Supporting Data

The data sets for draft genomes of *L. ferriphilum* DX (MPOJ00000000) and ZJ (MPOK00000000) were available in the NCBI repository. The versions described in this paper were version MPOJ02000000 and MPOK02000000, respectively.

## Results

### General Features of *L. ferriphilum* Genomes

A summary of the features of each *L. ferriphilum* genome was shown in **Table 1**. We listed the essential characteristics, such as genome size, GC content, and the number of predicted protein-coding sequences (CDSs). Generally, the five genomes varied in size (ranging from 2.33 to 2.48 Mbp) with the number of CDSs ranging from 2,273 to 2,419 (excluding RNA genes), indicating an intra-species variation. The CheckM program (Parks et al., 2015) was employed to estimate the completeness of draft genome of *L. ferriphilum* isolates, suggesting the high values of genome completeness (≥90.42%). Quality estimates of genomes based on collocated marker genes exhibit a bias, leading to an overestimated completeness. As stated by Parks et al. (2015), nevertheless, bias correction could be approximate owing to confounding factors such as gene collocation so long as the observed genomes are substantially complete (>70%). Furthermore, *L. ferriphilum* Sp-Cl has larger genome, but the GC content is slight different compared to its counterparts. All strains harbor many tRNA genes (ranging from 48 to 53) that cover all the 20 amino acids. While the majority of CDSs (ranging from 70.9 to 74.3%) in individual genomes could be assigned to COG categories, the remaining CDSs showed no sequence identity to any previously reported sequence. Of all isolates, functional analysis based on COG classification revealed that the five most abundant function categories are ‘Cell wall/membrane/envelope biogenesis [M],’ ‘Energy production and conversion [C],’ ‘Amino acid transport and metabolism [E],’ ‘Replication, recombination, and repair [L],’ and ‘Translation, ribosomal structure, and biogenesis [J]’ (Supplementary Table S2).

### Genome-to-Genome Distance Calculation

**FIGURE 1:**
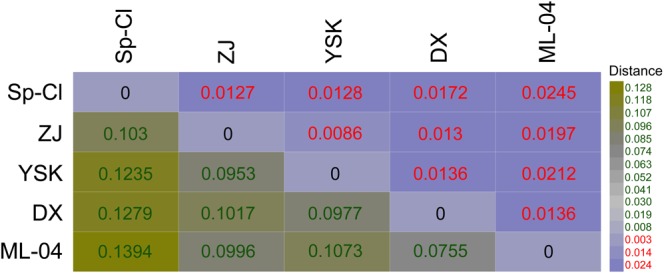
Distance phylogeny depicting the potential genome-genome distance by paired comparison using the online platform GGDC v2.1.

We inferred the genome-content-based distance matrix using a digital DDH approach. GGDC analysis showed a summary of strain-to-strain comparisons of *L. ferriphilum* genomes, suggesting that the strain-to-strain distances varied in genome content (**Figure 1**). Based on the paired comparisons, genomic variation among bacterial strains in some instances might be expected to comply with environmental heterogeneity and geographic distributions. For instance, distance phylogeny mirrored that strain Sp-Cl, which was isolated from leaching solutions draining from the bioleaching heap at Spence mine (Issotta et al., 2016), was more distantly associated with the others analyzed in this study. Strains ZJ, DX, and YSK, isolated from bioleaching environments at Dexing copper mine, were most closely related to each other.

### Core and Pan-Genome Analysis

**FIGURE 2:**
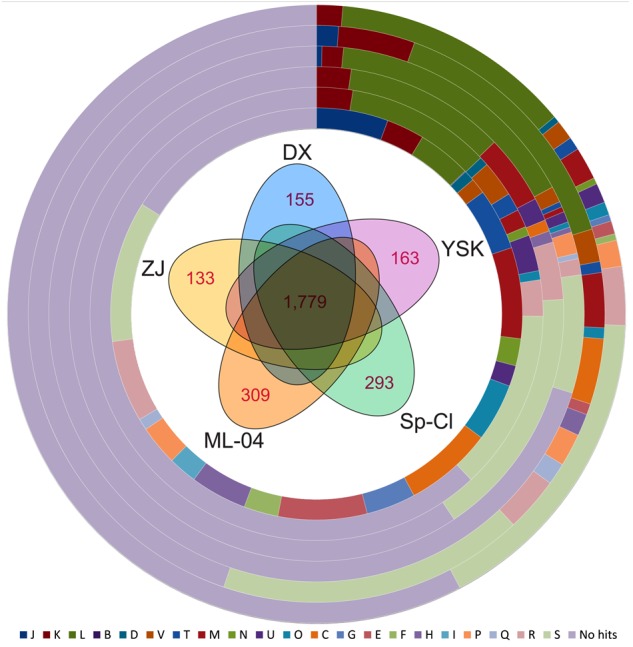
Pan-genome analysis of *L. ferriphilum* isolates. Venn diagram showing the core genome shared by all strains and strain-specific genes unique in individual genomes are indicated in the figure center. The percentages of core genes, DX-specific genes, ZJ-specific genes, ML-04-specific genes, YSK-specific genes, and Sp-Cl-specific genes assigned to COG classification are shown on the 1st to 6th ring from the inside. Detailed description for COG categories are provided in Supplementary Table S2.

We further performed pan-genome analysis to identify corresponding core and dispensable genome. A total of 3,455 predicted CDSs were found in the genomes of five *L. ferriphilum* strains and grouped into 2,402 homologous gene clusters. Further inspection uncovered that a core genome containing 1,779 putative CDSs was identified in *L. ferriphilum* species using a five-way best-match BLASTP search (**Figure 2**). This core genome represented 74 to 78% of proteome within each strain, illustrating a relatively high degree of genomic diversity compared to other bacterial groups such as *Erwinia amylovora* (Mann et al., 2013). Core genome encodes proteins that are responsible for fundamental housekeeping functions (Loper et al., 2012), and dispensable genome mainly contributes to species diversity and confers selective advantages (Medini et al., 2005; Tettelin et al., 2008; Vernikos et al., 2015). As expected, the vast majority of genes that are essential to the basic lifestyle of the species made up the core genome. Of the 1,779 core genes, relatively high percentage of CDSs were predicted to be assigned to COG categories [M] (7.14%), [E] (6.97%), [C] (6.91%), and [J] (5.62%) based on functional annotation (**Figure 2**).

Apart from the core genome, these dispensable genes contain strain-specific genes and genes shared by a subset of *L. ferriphilum* genomes. Pairwise comparisons provide insights into the strain-specific genes that are unique in each genome. Functional analysis by means of COG classification showed that the abundant genes only present in individual genomes were assigned to COG category [L], compared to that in core genome.

### Modeling the Expansion of *L. ferriphilum* Pan-Genome

**FIGURE 3:**
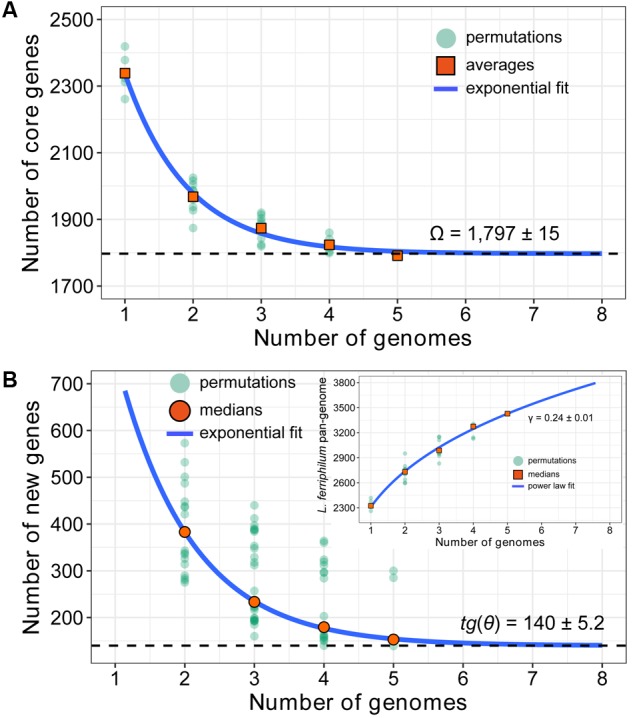
Mathematical modeling of *L. ferriphilum* pan-genome estimating the sizes of core genes **(A)**, new genes and pan-genome **(B)**. More details for modeling approaches are presented in Section “Extrapolation Models for *L. ferriphilum* Pan-genome.”

In theory, new genes would expand the genome of the species, as novel strain is sequenced (Medini et al., 2005). Accordingly, a mathematical extrapolation based on the available data might provide an opportunity to estimate the sizes of core genes and pan-genome of bacterial species. Counting CDSs only, a large number of pan-genome (a total of 3,455 CDSs in five strains) includes 1,779 core genes and 1,676 dispensable genes. Each of the five genomes of *L. ferriphilum* species contains 133 to 309 CDSs (6 to 13% of the predicted proteome) that are unique in respective strain (**Figure 2**). Based on mathematical modeling, the genomic dataset at hand was used to further predict the estimated number of additional genes that might be available to fully characterize intra-species diversity (**Figure 3**).

The predicted number of core genes with sequential inclusion of each new sequenced genome was extrapolated by fitting the exponential decay function *F_c_* = *κ_c_*exp(-*n*/*τ_c_*) + Ω (Tettelin et al., 2005). The resulting permutations of step-wise addition for each of the five genomes were shown, and the average counts were taken on the size of core genome. As depicted in **Figure 3A**, the number of core genes shared by all observed strains initially decreased with the addition of new genome. The extrapolated curve following a steep slope reached a minimum of 1,797 [mean ± standard deviation (SD): 1,797 ± 15] genes after the 5th genome was included (Supplementary Table S3). As predicted by exponential regression model, the number of core genes, which are conserved genes universally present in all considered strains (Zhang and Sievert, 2014), was relatively constant, and the additional genome added would not expected to significantly affect its size.

The number of new genes added by novel sequenced genome could be examined by fitting a decaying exponential to determine the expansion of *L. ferriphilum* pan-genome. The ‘open’ or ‘closed’ pan-genome within a given bacterial species was mathematically evaluated by fitting exponential regression model (Tettelin et al., 2008). An ‘open’ pan-genome has a large and undetermined number of additional genes, and its size would increase unboundedly with the number of sample strains. In contrast, the size of ‘closed’ pan-genome would quickly saturate to a limiting value after a certain number of sequenced genomes are added, suggesting that novel sequenced genome could not expand species’ pan-genome (Tettelin et al., 2008; Zhang and Sievert, 2014; Vernikos et al., 2015). In our study, the resulting extrapolation showed that the number of new genes was relatively large, and this number decreased to 140 (median ± SD: 140 ± 5.2) after the 5th genome was included (**Figure 3B** and Supplementary Table S3). In other words, a non-zero asymptotic value (140) of additional strain-specific genes would be added when novel genome was sequenced, leading to an ‘open’ pan-genome. Furthermore, a power law regression function *P_s_* = *κn*^γ^ revealed the *L. ferriphilum* pan-genome with an average parameter (γ) of 0.24 (median ± SD: 0.24 ± 0.01; Supplementary Table S3). For 0 < γ < 1, the pan-genome is open (Tettelin et al., 2008). That was equivalent to say that the size of *L. ferriphilum* pan-genome followed the Heaps’ law (Heaps, 1978) and was increasing and unbounded with the inclusion of novel genomes.

### Identification of Potential Mobile Genetic Elements

Mobile genetic elements are defined as specific genome segments, which encode for putative functions related to intra- and extracellular movement of DNA, and are regarded to be signatures of HGT events (Loper et al., 2012; Ullrich et al., 2016). In this study, MGEs including transposases, integrases, and phage-associated genes were identified and compared in all five *L. ferriphilum* genomes. In addition, genomic islands (GIs) and CRISPR/Cas systems (clustered regularly interspaced palindromic repeats/CRISPR-associated genes) were taken into account.

Transposases and integrases in *L. ferriphilum* isolates were predicted using ISFinder (Supplementary Table S4). The number of transposases per strain ranged from 63 (ZJ) to 106 (ML-04). While members of the IS1595, IS21, ISL3, and Tn3 families were most common, there were also IS classes that were only present in individual genomes; such as IS1 in Sp-Cl.

Except for transposons, the genomes harbored 3 to 15 GIs ranging from 6 to 82 kbp in size (Supplementary Table S5). In prokaryotic genomes, GIs are defined as the clusters of genes that contain integrative conjugative elements, prophages, integrons, conjugative transposons, and integrated plasmids (Langille et al., 2010). GIs carried significant cargo genes that potentially related to certain selective advantages, such as virulence and drug resistance, and might increase ecological fitness (Whittle et al., 2002; Mavrodi et al., 2009; Seth-Smith and Croucher, 2009). In general, the five genomes in our study were predicted to harbor 29 GIs, and the number of GIs in ML-04 (15) was much more than those in others. As is common in most bacteria, numerous genes within these GIs were annotated as hypothetical protein, suggesting that biological functions of these elements still need to be explored. Among these cargo genes of GIs, some were likely to encode predicted functions that were related to restriction-modification systems (GI3 and GI4 in ML-04), transcriptional regulators (GI2 and GI3 in DX; GI6, GI7, GI10, GI11, GI12, GI14, and GI15 in ML-04), assorted transporters (GI6, GI13, GI14, and GI15 in ML-04; GI1 in Sp-Cl), signal transduction protein (GI9 in ML-04), acetylglutamate kinase (GI1 in DX; GI1 in ZJ; GI1 in ML-04; GI1 in YSK; GI2 in Sp-Cl), and secretion systems (GI6, GI7, and GI13 in ML-04). Several GIs also included IS family transposases (GI2 and GI4 in DX; GI3, GI4, GI5, GI6, and GI9–GI14 in ML-04; GI2 and GI4 in YSK; GI3 in Sp-Cl) and phage integrase family proteins (GI1 and GI8 in ML-04; GI1 in YSK; GI2 in Sp-Cl).

CRISPR (clustered regularly interspaced short palindromic repeats), which occur in many bacterial and archaeal genomes, are responsible for prokaryotic immunity to the invasion of phages and plasmids (Loper et al., 2012; Plagens et al., 2015; Ullrich et al., 2016). In strains DX, ML-04, and YSK, putative CRISPR identified by the CRISPRFinder server (Grissa et al., 2007) were present within the called genes, instead of in these intergenic regions. Besides, the predicted repetitive elements were not contiguous to genes that potentially encode typical CRISPR-associated proteins, which were necessary for CRISPR functionality. Conversely, *L. ferriphilum* strains Sp-Cl and ZJ were predicted to harbor a couple of CRISPR system, in which the palindromic repeats consist of a repeat-spacer array, immediately upstream/downstream of one/two *cas* genes (e.g., *cas1, cas2*, and *cas6*) or other CRISPR-associated genes (e.g., *csf2* and *cse3*; Supplementary Figure S2). Based on comparison to CRISPR/Cas systems in other bacteria (Makarova et al., 2011), the related systems in Sp-Cl and ZJ were classified to be type I-E (Supplementary Table S6), which were reported to target foreign DNA. The CRISPR/Cas systems present in Sp-Cl and ZJ strongly suggested that the co-evolution occurring both phage and host was an important mechanism that might drive adaptive evolution of bacterial genome.

### Comparison of Genome Architectures Highlighting Specific Genomic Regions of Interest

BLASTN-based whole genome comparisons were performed and visualized using the Circos software (Supplementary Figures S3A–E). On the whole, the presence or absence of genome segments visually revealed the intra-species diversification of *L. ferriphilum* isolates at genomic level. In this context, 14 sections from corresponding genomes were further investigated by means of pairwise alignment and manual annotation.

Using the draft genome of strain DX as reference for the BLASTN-based genome comparison, many genomic regions unique in this strain were identified (Supplementary Figure S3A). Further inspection showed that a large cluster (approximate 16 kbp) on the contig11 was predicted to harbor 19 genes, most of which were annotated as hypothetical proteins (Supplementary Table S7). Intriguingly, two genes encoding putative type VI secretion-associated proteins were found to be located in this region. Type IV secretion system (T4SS) is a large protein complex, which has been regard to be the signature of conjugative DNA transfer (Rêgo et al., 2010; Wallden et al., 2010; Trokter et al., 2014). Likewise, T4SS-associated proteins were also predicted in other genomic regions from various strains. Especially, a nearly complete set of Dot/Icm secretion system, which is belonging to T4SS, were detected in Sp-Cl genome (sections 11 in Supplementary Figure S3E and Supplementary Table S7). In addition, a series of genes encoding putative type IV pilus biogenesis proteins were predicted to be distributed on contig11 in strain Sp-Cl (Supplementary Table S7). As is reported, type IV pili facilitate the adhesion of microbial cell on mineral surfaces (Jin et al., 2011), thereby providing a reaction space between microbial strains and mineral surface. Additionally, many unique regions in a subset of genomes were identified by pairwise comparison (Supplementary Table S7). These genome segments were predicted to harbor a plenty of genes, although most of them were annotated as hypothetical proteins with unidentified functions. Further investigation showed that a collection of HGT signatures, including putative phage-associated genes and transposases, were dispersed in the neighborhood of the aforementioned genes. Accordingly, we inferred that these genes with certain functions might be introduced via HGT events.

**FIGURE 4:**
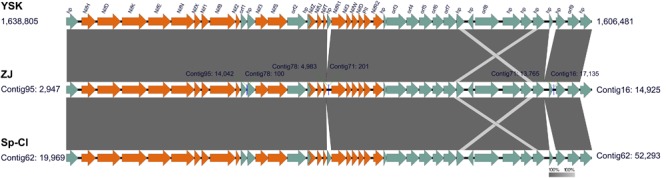
Predicted genomic segments potentially associated with nitrogen fixation in the genomes of *L. ferriphilum* strains ZJ, YSK, and Sp-Cl. The putative *nif*-genes (orange) are indicated using the software EasyFig, and the others include genes encoding hypothetical proteins (hp) and proteins with identified functions. Genomic loci of all analyzed genes are also given. More details are presented in Supplementary Table S7.

In the genomes of *L. ferriphilum* strains except for DX and ML-04, notably, a large cluster (approximate 32 kbp) harboring 42 genes was identified to be potentially associated with nitrogen fixation (**Figure 4**). Pairwise comparison of potential nitrogen-fixing gene cluster in *L. ferriphilum* isolates using EasyFig was attempted to demonstrate the identical gene content, order, orientation, and high nucleotide sequence identity of the 42 genes. As stated by Baker and Banfield (2003), the fixation of externally-derived nitrogen in extremely low pH environments was difficult to be directly observed, thus nitrogen fixation in these settings was enigmatic. However, previous studies documented that *Leptospirillum* groups including *L. ferriphilum* have been shown to possess a diazotrophic lifestyle (Parro and Moreno-Paz, 2004; Tyson et al., 2005; Goltsman et al., 2009, 2013; Galleguillos et al., 2011, 2013). Very recently, a complete genome of *L. ferriphilum* DSM 14647^T^ was acquired in virtue of re-sequencing, and a previously undiscovered nitrogenase cluster for N_2_ fixation was reported (Christel et al., 2018). In our study, however, *nif*-associated genes encoding putative nitrogenase structural subunits NifHDK, MoFe cofactor biosynthesis proteins NifENX, and various additional subunits were absent in *L. ferriphilum* strains DX and ML-04 (Supplementary Table S7). Furthermore, signatures of HGT were detected in order to infer the potential origin of genes involved in nitrogen fixation. In strains ZJ, YSK, and Sp-Cl, however, no putative transposases, integrases, as well as phage-associated genes were predicted in the genomic neighborhoods. We thus interpreted this as an indication that *nif* -associated genes in the genomes of *L. ferriphilum* strains ZJ, YSK, and Sp-Cl might be inherited from a common ancestor, while the absence of homologous genes in *L. ferriphilum* strains DX and ML-04 was more likely the result of gene loss rather than gene gain caused by the event of HGT.

## Discussion

In *Bacillus anthracis*, the number of new genes rapidly converges to zero after the addition of fourth sequenced genome, and the pan-genome size quickly saturates to a limiting value (Medini et al., 2005). Thus, only four genome sequences might be sufficient to characterize the pan-genome of this species well. In terms of *Staphylococcus aureus* strains species, a prediction model was observed in the study of 17 existing genomes (Boissy et al., 2011). In this case, the number of new genes added to the pan-genome tends to zero until the 30th predicted genome is added, indicating a ‘closed’ pan-genome. According to the Heaps’ law model, a threshold parameter (0 < γ < 1) in our mathematical modeling suggested that the pan-genome of *L. ferriphilum* is ‘open.’ As an ‘open’ microbial pan-genome, species colonizing multiple environments may exchange genetic material with the others in multiple ways (Medini et al., 2005; Tettelin et al., 2008), resulting in the emergence of additional genes with novel sequenced genomes and thus enlarging the gene repertoire of species. Compared to other species with an ‘open’ pan-genome, such as *E. amylovora* (Mann et al., 2013), *L. ferriphilum* species has relatively more dispensable genes, indicating the higher genome plasticity via genetic exchange during evolution. In contrast to other species such as *Buchnera aphidicola* with a low capacity to acquire alien genes (Medini et al., 2005), especially, the introduction of novel genes might contribute to fascinating discoveries of novel traits of *L. ferriphilum* isolates.

The acquisition of genes, often accompanied by HGT events, and the loss of genes and genome segments are the two main mechanisms that drive adaptive evolution of microbial genomes (Gogarten et al., 2002; Boon et al., 2014; Albalat and Cañestro, 2016). MGEs including transposases, integrases, and phage-associated genes are generally regarded as indicators of HGT (Waack et al., 2006; Juhas et al., 2009; Acuña et al., 2013; Ullrich et al., 2016). In this study, various MGEs were predicted and classified in the five *L. ferriphilum* genomes (see section “Identification of Potential Mobile Genetic Elements”). Notably, the investigation of unique genomic regions in a subset of genomes revealed that signatures of HGT were predicted to be located in the neighborhood of these observed genomic regions, suggesting that *L. ferriphilum* genomes might undergo several events of rearrangements and HGT to recruit the novel genes with certain functions. Species inhabiting isolated econiches with limited access to the global gene pool of microorganisms have few opportunities for the acquisition of foreign genes (Medini et al., 2005). By contrast, *L. ferriphilum* species was predicted to have an ‘open’ pan-genome, indicating the capacity to introduce alien genes by genetic exchanges with other community members in the common microhabitats. The identification of MGEs, especially plentiful phage-associated genes, further suggested that HGT might play a critical role in bacteria-phage co-evolution and speciation of *L. ferriphilum* strains. Collectively, extensive gene recruitment via HGT has extended the genomic intra-species diversity, suggesting plentiful lateral exchange of genetic material as a high-efficient adaptive strategy in these adverse environments.

Comparisons of genome architectures of five *L. ferriphilum* strains revealed that nitrogen-fixing gene cluster in strains ZJ, YSK, and Sp-Cl was likely to be originally derived from a common ancestor, while the *nif*-genes might be lost in strains DX and ML-04. In the latter two strains, apparently, the incapacitation of nitrogen fixation via gene loss has profoundly contributed to shaping their metabolic profiles and to reducing their genome size. In some organisms, genome reduction could be explained by the Black Queen Hypothesis, a theory that seeks to demonstrate the community-dependent adaptation (Morris et al., 2012). In free-living organisms, genome reduction may leave them dependent on co-occurring members of microbial community for lost metabolic functions. And the loss of certain dispensable functions in individual members became beneficial as long as the production of metabolic function is just sufficient to support the entire community. Many studies revealed that acidophilic prokaryotes including *Acidithiobacillus* and *Leptospirillum* spp. ubiquitously occurred in extremely acidic environments such as acid mine drainage (Breuker et al., 2009; Chen et al., 2013; Zhang et al., 2016c,d). However, nitrogen fixation in these settings seems to be partitioned into a small fraction of microbial members in a common community (Méndez-García et al., 2015), such as *Acidithiobacillus ferrooxidans* (Valdés et al., 2008) and *L. ferrodiazotrophum* (Tyson et al., 2004). In largely aerobic and microaerophilic acidic environments, molecular oxygen hinders the activity of nitrogenase. Instead, *A. ferrooxidans* may circumvent this barrier by using the electron donor tetrathionate and electron acceptor ferric iron (rather than O_2_) for nitrogen fixation (Norris et al., 1995; Baker and Banfield, 2003). In this case, these diazotrophs including *A. ferrooxidans* may more effectively fix the environmental nitrogen, and then provide alternative nitrogen compounds for the growth of other co-existing species without nitrogen-fixing ability. Besides, more than one strain belonging to the same species could be co-occurring in certain environments or industrial processes (Remonsellez et al., 2009; He et al., 2010; Mutch et al., 2010). Accordingly, it was inferred that some strains of *L. ferriphilum* species harboring the ability of nitrogen fixation co-exist in a common community, and could contribute by initially fixing nitrogen to support the nitrogen supply of whole community (including homologous strains lacking *nif* genes and other members of the microbial community). As for *L. ferriphilum* strains DX and ML-04, it appears to be a way to compromise via losing the ability of nitrogen fixation in the context of sufficient public goods (nitrogen compounds) produced by other diazotrophic members in the common community. This ‘compromise’ to some extents, may be selectively favored by reducing their nutrient requirements and further economizing the limiting resources of the whole microbial community.

Extrapolation modeling revealed that *L. ferriphilum* species was predicted to harbor an ‘open’ pan-genome. That is, more novel genes were introduced to offset the ‘abandon’ genes after new *L. ferriphilum* genomes were sequenced. Since the introduction of new genes is observed to be accompanied by a parallel abandonment of some other ones, it is likely that trade-off between environmental adaptation and biological fitness might drive the evolution of *L. ferriphilum* genomes. In other words, the recruitment of novel genes potentially related to species-specific adaptation might contribute to the selective abandonment of some genes that are likely to be redundant in an exclusively biotrophic lifestyle, probably driving the adaptive evolution of *L. ferriphilum* species. An intriguing study was that some mechanisms within *Saccharomyces paradoxus* have evolved to compensate for the fitness cost of improving cadmium resistance (Chang and Leu, 2011). Other studies on antibiotic resistance of microorganisms exhibited that the fitness cost on these mechanisms is accompanied by a parallel reduction of biological fitness, such as substrate utilization (Kang and Park, 2010) or bacterial growth rate (Andersson and Hughes, 2010). Collectively, findings presented here imply that *L. ferriphilum* genomes might make sacrifices for the improvement of adaptive evolution via subordinating certain biological functions.

## Conclusion

In *L. ferriphilum* species with an ‘open’ pan-genome, novel genes lead to the expansion of its gene repertoire after multiple genomes were sequenced. The introduction of new genes by genetic material exchange in multiple ways such as HGT might be a crucial evolutionary force of microbial species to respond to the external environmental perturbations. Furthermore, the recruitment of new genes was observed to be accompanied by a parallel abandonment of some other genes. In other words, the fitness cost of improving environmental adaptation might drive the evolution of *L. ferriphilum* genomes. Taken together, the findings advance our understanding of evolutionary strategies of *L. ferriphilum* genomes, and further provide robust evidences for the potential relatedness between hereditary variation of *L. ferriphilum* genomes with its adaptive evolution.

## Author Contributions

XZ drafted and wrote the manuscript. XL, FY, and LC participated in the discussion.

## Conflict of Interest Statement

The authors declare that the research was conducted in the absence of any commercial or financial relationships that could be construed as a potential conflict of interest.

## Footnotes

1 http://ggdc.dsmz.de/distcalc2.php

2 http://www.genome.jp/tools/blast/
