# Supplementary material for: Pan-Genome Analysis Links the Hereditary Variation of Leptospirillum ferriphilum With Its Evolutionary Adaptation
Source: Front Microbiol. 2018 Mar 27;9:577. doi: 10.3389/fmicb.2018.00577 (PMC5880901; doi:10.3389/fmicb.2018.00577)
Supplement: Supplementary file 1 [file Data_Sheet_1.pdf]

Supplemental Material for

Pan-genome analysis links the hereditary variation of *Leptospirillum ferriphilum* with its evolutionary adaptation

Xian Zhang,[1] Xueduan Liu,[2,3] Fei Yang,[1] Lv Chen [1]*

[*]**Corresponding author:** Lv Chen

[1]*Department of Occupational and Environmental Health, Xiangya School of Public Health, Central South University, Changsha, China;*

[2]*School of Minerals Processing and Bioengineering, Central South University, Changsha, China;*

[3]*Key Laboratory of Biometallurgy of Ministry of Education, Central South University, Changsha, China*

SUPPLEMENTARY FIGURES

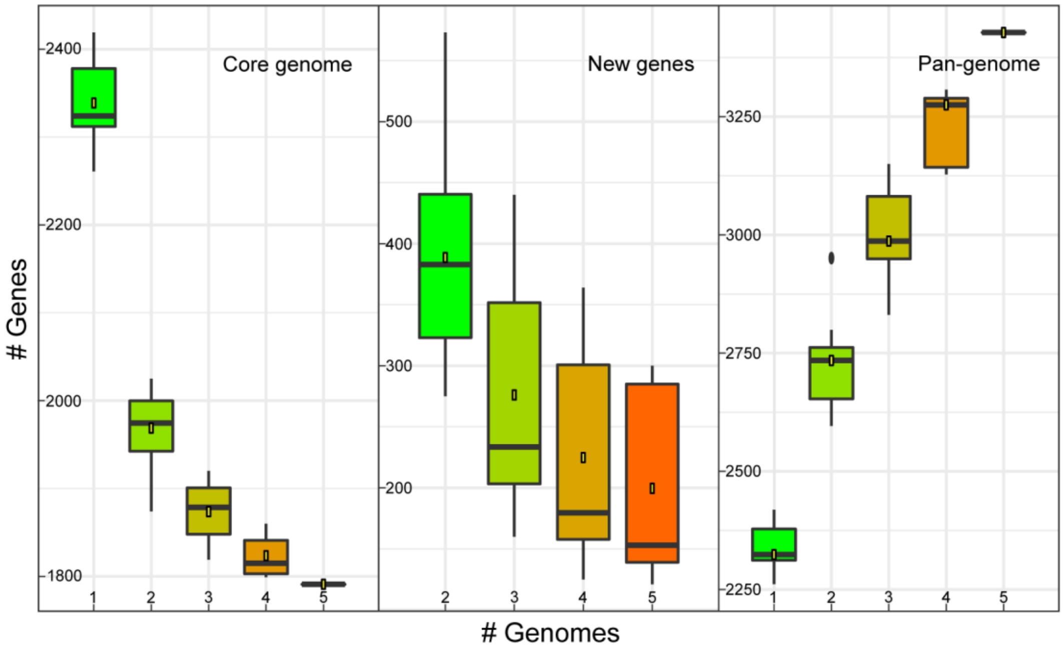

FIGURE S1 Distributions of core genome, new genes, and pan-genome' values: averages vs. medians. Dark spots represent the distributions of values of core genome, new genes, and pan-genome. For each value of N, a box-plot of the distribution indicating the median (black lines) and the average (yellow rectangle) are given.

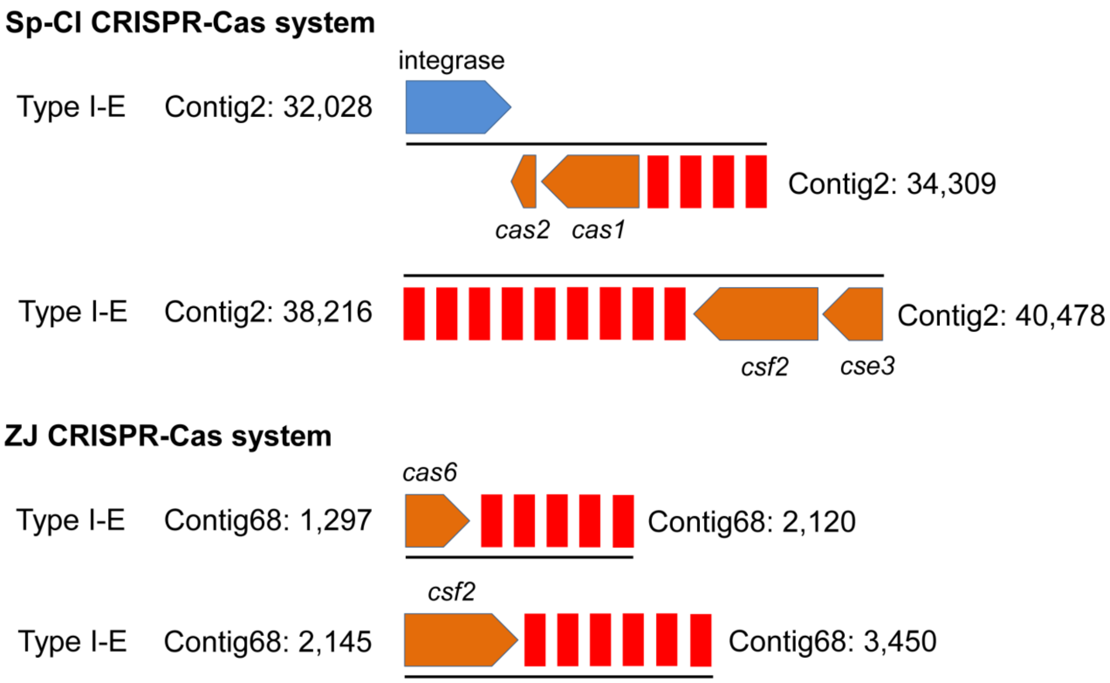

FIGURE S2 Predicted genes involved in CRISPR/Cas systems in the genomes of *L. ferriphilum* strains Sp-Cl and ZJ. Putative CRISPRs are shown as red rectangles.

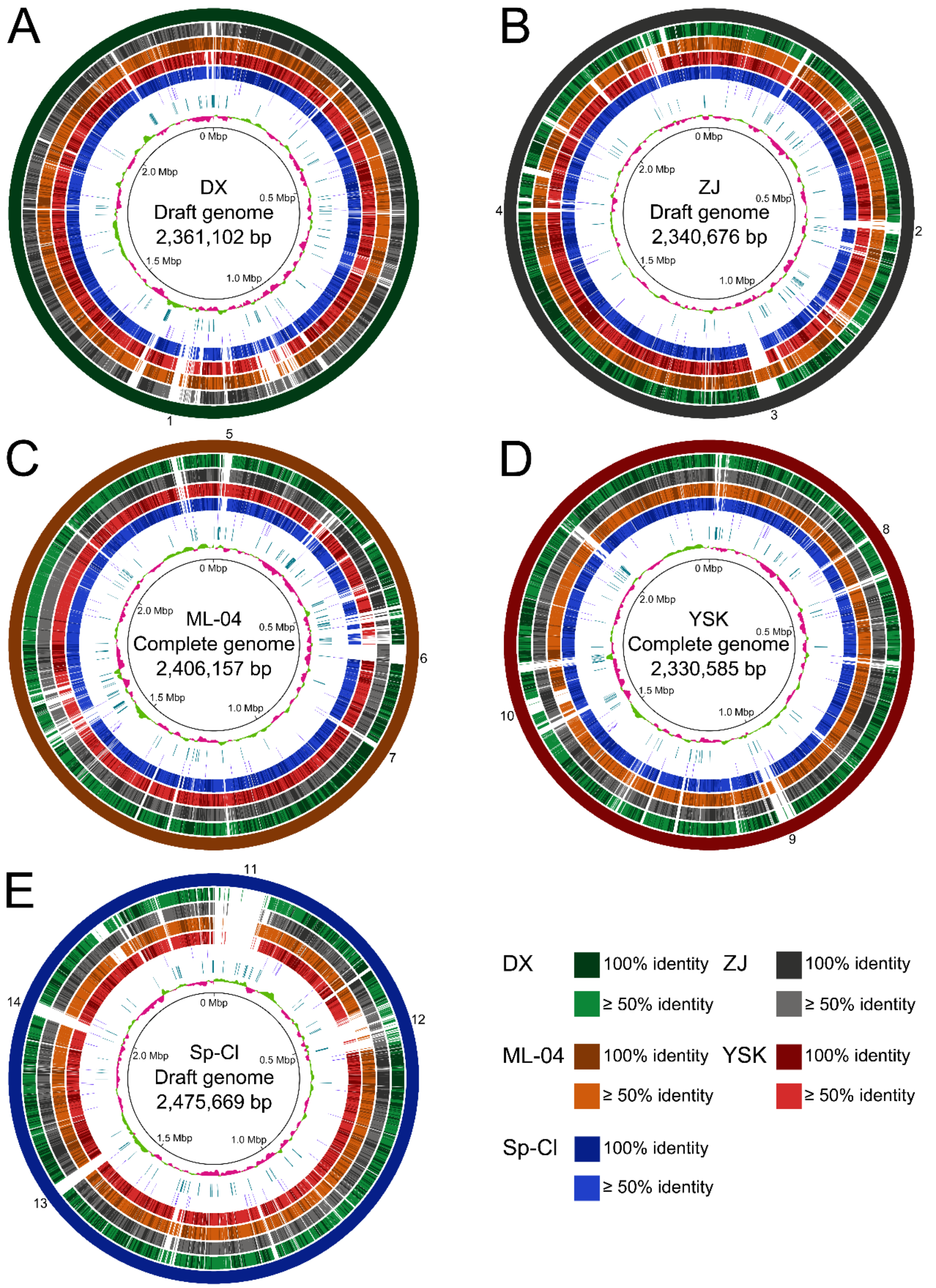

FIGURE S3 Reference-based whole genome comparisons of the five *L. ferriphilum* strains. Each of the five genomes was used as a reference in turn to perform the Blastn-based whole genome comparison. All reference genomes and their sizes are indicated in the figure center, including DX (A), ZJ (B), ML-04 (C), YSK (D), and Sp-CI (E). For each reference genome, G+C content, transposases, and tRNA are indicated

on the 1th to 3th ring from the inside. Besides, various rings in different colors represent individual genomes.

SUPPLEMENTARY TABLES

TABLE S1 Normality Test using Shapiro-Wilk method.

Items	Numbers of genomes	W	P-value
Core genome	1	0.9778	0.923
	2	0.9481	0.646
	3	0.9367	0.517
	4	0.9049	0.438
New genes	2	0.9428	0.271
	3	0.8777	0.002
	4	0.8450	0.004
	5	0.8090	0.957
Pan-genome	1	0.9778	0.923
	2	0.9217	0.372
	3	0.9148	0.316
	4	0.8090	0.096

TABLE S2 The distribution of the COG functional classes in the genomes of *L. ferriphilum* isolates.

COG category	Description	DX	ZJ	ML-04	YSK	Sp-Cl
B	Chromatin structure and dynamics	1	1	1	1	1
C	Energy production and conversion	**133**	**138**	**137**	**142**	**134**
D	Cell cycle control, cell division, chromosome partitioning	17	17	15	15	15
E	Amino acid transport and metabolism	**125**	**129**	**124**	**126**	**130**
F	Nucleotide transport and metabolism	47	47	46	46	47
G	Carbohydrate transport and metabolism	70	69	74	70	68
H	Coenzyme transport and metabolism	79	79	79	80	79
I	Lipid transport and metabolism	42	41	42	43	40
J	Translation, ribosomal structure, and biogenesis	**113**	**113**	**107**	**117**	**110**
K	Transcription	65	57	63	67	61
L	Replication, recombination, and repair	**116**	**120**	**158**	**120**	**123**
M	Cell wall/membrane/envelope biogenesis	**149**	**144**	**156**	**145**	**151**
N	Cell motility	44	43	38	39	43
O	Posttranslational modification, protein turnover, chaperones	83	81	80	79	81
P	Inorganic ion transport and metabolism	64	62	70	67	65
Q	Secondary metabolites biosynthesis, transport and catabolism	17	18	18	19	17
R	General function prediction only	127	127	133	126	121
S	Function unknown	249	272	256	243	278
T	Signal transduction mechanisms	89	93	88	83	90
U	Intracellular trafficking, secretion, and vesicular transport	33	34	31	29	33
V	Defense mechanisms	32	28	30	32	27
	No hits	629	599	632	584	705

Except for COG categories [S] and [R], the five most abundant COG categories are shown in bold.

TABLE S3 The fitting results of function F_c (A), F_s (B), and P_s (C) for *L. ferriphilum* strains.

**(i)**				
κ_c	τ_c	**Ω**	**R-square**	**Adj. R-square**[2]
1,607 ± 184***	0.91 ± 0.10***	1,797 ± 15***	0.9971	0.9943
**(ii)**				
ε_s	τ_s	$tg(\theta)$	**R-square**	**Adj. R-square**[2]
1,591± 16***	1.06 ± 0.07***	140 ± 5.2***	0.9997	0.9991
**(iii)**				
**κ**	**γ**		**R-square**	**Adj. R-square**[2]
2,311 ± 24***	0.24 ± 0.01***		0.9968	0.9936

The parameter results are in form of estimate ± standard error, and labeled by '***' when $p < 0.001$.

1. The number of strains

2. Adjust R-square = 1-(1-R-square)*(n-1)/(n-k), where n is the number of strains, and k is the number of parameter (here, k=3).

TABLE S4 Predicted insertion sequence elements in the *L. ferriphilum* genomes.

**IS family**	**DX**	**ZJ**	**ML-04**	**YSK**	**Sp-Cl**
IS1	0	0	0	0	1
IS110	2	0	**12**	1	3
IS1182	0	2	0	2	1
IS1380	1	4	0	2	0
IS1595	6	**6**	**12**	**8**	**7**
IS1634	1	0	4	1	2
IS200/IS605	0	0	0	1	1
IS21	**7**	**5**	**18**	4	5
IS256	5	4	**15**	**8**	4
IS3	2	4	0	6	3
IS4	2	2	2	2	1
IS481	1	1	1	1	1
IS5	3	3	3	2	2
IS607	2	2	2	3	1
IS630	2	3	2	4	1
IS66	**7**	**8**	5	**7**	**7**
IS91	6	4	9	2	5
ISKra4	0	1	1	2	3
ISL3	**12**	**8**	11	**12**	**10**
ISNCY	1	1	1	0	0
Tn3	**9**	**5**	8	6	**6**
Total	69	63	106	74	64

The four most abundant CDS potentially related to IS family are highlighted in bold.

TABLE S6 The putative CRISPR (clustered regularly interspaced short palindromic repeats) in the genomes of strains Sp-Cl and ZJ.

Strain	Type	Strand	*cas* genes	Repeats sequence	Length	Copy	Spacer length
**Sp-Cl**	type I-E	–	*cas2-cas1*	CCTTCACCCCCACATACGTGGGGACTAC	28	4	33
	type I-E	–	*csf2-cse3*	CCTTCACCCCCACATACGTGGGGACTAC	28	9	33
**ZJ**	type I-E	+	*cas6*	GTATTCCCCGCGCACGCGGGGGTGAAAC	28	5	33
	type I-E	+	*csf2*	GTATTCCCCGCGCACGCGGGGGTGAAAC	28	6	33
